# Does motor imagery share neural networks with executed movement: a multivariate fMRI analysis

**DOI:** 10.3389/fnhum.2013.00564

**Published:** 2013-09-12

**Authors:** Nikhil Sharma, Jean-Claude Baron

**Affiliations:** Department of Clinical Neuroscience, University of CambridgeCambridge, UK; Inserm U894, Centre Hospitalier Sainte-Anne, Sorbonne Paris CitéParis, France

**Keywords:** motor imagery, functional imaging, fMRI, mental imagery, brain mapping

## Abstract

**Introduction:** Motor imagery (MI) is the mental rehearsal of a motor first person action-representation. There is interest in using MI to access the motor network after stroke. Conventional fMRI modeling has shown that MI and executed movement (EM) activate similar cortical areas but it remains unknown whether they share cortical networks. Proving this is central to using MI to access the motor network and as a form of motor training. Here we use multivariate analysis (tensor independent component analysis-TICA) to map the array of neural networks involved during MI and EM.

**Methods:** Fifteen right-handed healthy volunteers (mean-age 28.4 years) were recruited and screened for their ability to carry out MI (Chaotic MI Assessment). fMRI consisted of an auditory-paced (1 Hz) right hand finger-thumb opposition sequence (2,3,4,5; 2…) with two separate runs acquired (MI & rest and EM & rest: block design). No distinction was made between MI and EM until the final stage of processing. This allowed TICA to identify independent-components (IC) that are common or distinct to both tasks with no prior assumptions.

**Results:** TICA defined 52 ICs. Non-significant ICs and those representing artifact were excluded. Components in which the subject scores were significantly different to zero (for either EM or MI) were included. Seven IC remained. There were IC's shared between EM and MI involving the contralateral BA4, PMd, parietal areas and SMA. IC's exclusive to EM involved the contralateral BA4, S1 and ipsilateral cerebellum whereas the IC related exclusively to MI involved ipsilateral BA4 and PMd.

**Conclusion:** In addition to networks specific to each task indicating a degree of independence, we formally demonstrate here for the first time that MI and EM share cortical networks. This significantly strengthens the rationale for using MI to access the motor networks, but the results also highlight important differences.

## Introduction

Athletes have used motor imagery (MI) for decades but recently there has been considerable interest in applying it to the patient population (Braun et al., 2006; Sharma et al., 2006). The general premise is that MI can be used as a surrogate for movement when a disease limits performance, for instance using MI training after stroke (Braun et al., 2006; Sharma et al., 2006; Ietswaart et al., 2011) or Parkinson's Disease (Heremans et al., 2012). The central assumption underlying this aproach is that MI and executed movement (EM) share neural substrates. Demonstrating that imagery and EM share neural substrates, rather than activate similar areas, would significantly enhance the rational for using MI training.

There are numerous behavioral studies that suggest MI and EM involve similar cognitive processes. For example the time taken to imagine a movement is similar to the time taken execute it (Decety et al., 1989). MI is confined by the same principles of motor control that govern EM. The reduction in accuracy with increasing speed (i.e., Fitt's Law) is maintained (Decety and Jeannerod, 1995) as is the asymmetry between dominant and non-dominant hand (Maruff et al., 1999). MI produces similar autonomic changes as EM, with significant increase in heart and respiratory rates (Jeannerod and Frak, 1999; Roure et al., 1999; Kazuo Oishi, 2000).

Given the strength of the behavioral studies it is perhaps not surprising that imaging studies regardless of the modality report that MI activates similar cortical regions to EM (Boecker et al., 2002; Lacourse et al., 2005; Hanakawa et al., 2008; Guillot et al., 2009). The cortical areas involved include the contralateral premotor areas, the primary motor cortex with some caveats, see (Sharma et al., 2008) as well as the cerebellum. These studies typically employed a massed univariate approach and have been useful in identifying significant differences between imagery and EM. For instance the contralateral primary motor cortex activation is both greater (Gerardin et al., 2000; Sharma et al., 2008) and topographically different (Sharma et al., 2008) during EM as compared to MI. The mass-univariate approach is less useful in concluding what neural substrates are common to each task. Generally this is inferred from involvement of similar cortical structures and a “lack of significant” difference when comparing tasks.

In this study we adopt a model-free approach using tensor independent component analysis to examine the cortical networks that are common to both MI and EM. Unlike the conventional mass univariate approach TICA is a powerful data driven approach capable of exploring similarities in cortical networks. A key aspect of this study is that MI and EM are treated as the same “blinded task” during the production of the independent-components (IC). In other words we make no prior assumptions as to the extent of overlap, if any, between the MI and EM. If the cognitive process of imagery and EM involve similar area but are actually distinct then the analysis will produce networks (i.e., IC) that relate to either MI or EM but not both. However, given the extensive behavioral literatures we hypothesize that three categories of networks will be identified; first, those networks that are present during EM only, which will involve the contralateral primary motor cortex; second, networks that are common to both MI and EM, involving premotor and posterior parietal areas; and finally networks that involve MI only, involving the premotor areas (Sharma et al., 2009a). Understanding which networks MI shares with EM will allow a richer understanding of how MI can be applied to the patient population with maximal effect.

## Methods

### Subjects

Fifteen healthy volunteers were recruited through local advertisement with a mean age of 28.4 years (*SD* = 6.2; 7 Male). Subjects overlapped with those reported in (Sharma et al., 2008) where we reported the differential involvement of BA4a and BA4p in MI and EM. They had no past medical history of any neurological, psychiatric or musculo-skeletal disorders and were not taking regular medication. All subjects were righted handed as assessed by the Edinburgh scale (Oldfeld, 1971)and gave written consent in accordance to the declaration of Helsinki and the protocol was approved by the Cambridge Regional Ethics Committee.

All subjects were assessed using the Chaotic Motor Imagery Assessment and were excluded if unable to perform IM adequately. The Chaotic Motor Imagery assessment is described briefly below, for a more detailed description see (Sharma et al., 2006, 2008, 2009a). During all tasks requiring explicit MI, subjects were given specific instructions to perform first person kinesthetic MI; not to view the scene from the 3rd person; and not to count or assign numbers or tones to each finger.

### Chaotic motor imagery assessment

Chaotic Motor Imagery is defined as an inability to perform MI accurately or, if having preserved accuracy, the demonstration of temporal uncoupling (Sharma et al., 2006). Briefly the CMIA consists of three components performed in the order they are described here.

First, subjects are shown 96 A4-sized picture cards of hands (4 different views, 12 rotations, left and right) and asked to identify whether the picture is of a left or right hand (Component 1). A score below 75% correct indicates that the subject is unable to perform accurate MI. Second, subjects are asked to perform MI of a finger sequence task (2,3,4,5,2..; Paced using Auditory cues at 1Hz; fMRI simulation Component 2). The duration of the finger tapping exercise varied and the subject had to confirm their position at the end of each block. Third, subjects are required to perform the same finger taping sequence initially using EM and then using MI (Component 3). During both phases of this test the external auditory pacing rate, which starts at 40 beats/min is increased by 10 beats every 5 s. The break point is defined as the time when the subject is unable to perform the task accurately. Subjects are excluded if the break point is greater for MI than for EM. During all tasks requiring MI, subjects were given specific instructions to perform first person MI; not to view the scene from the 3rd person; and not to count or assign numbers or tones to each finger. Subjects were excluded if unable to perform MI adequately.

### Functional MRI

#### Motor (imagery) paradigm

The fMRI used an established block design (Sharma et al., 2008, 2009b) that consists of auditory-paced (1 Hz) right hand finger-thumb opposition sequence (2,3,4,5, 2…) with two separate runs acquired (MI & rest and EM & rest). Subjects were instructed to keep their eyes closed throughout the session. We used individually calibrated bilateral fiber-optic gloves (Fifth Dimension Technologies, SA) to monitor finger movements, excluded inappropriate movement and to confirm the performance of MI—after each MI block subjects confirmed the finger they were currently imagining was the correct “stop finger” for the length of sequence (which varied). After scanning subjects were asked to rate the vividness of MI performance on a seven point scale (Alkadhi et al., 2005).

#### Data acquisition

A 3-Tesla Brucker MRI scanner was used to acquire both T2-weighted and proton density anatomical images and T2^*^-weighted MRI transverse echo-planar images sensitive to the BOLD signal for fMRI (64 × 64 × 23; FOV 20 × 20 × 115; 23 slices 4 mm, *TR* = 1.5 s, *TE* = 30 ms, Voxel Size 4 × 4 × 4).

#### Image analysis

Analysis was carried out using Tensorial Independent Component Analysis (Beckmann and Smith, 2005) as implemented in MELODIC (Multivariate Exploratory Linear Decomposition into IC) Version 3.09, part of FSL (FMRIB's Software Library, www.fmrib.ox.ac.uk/fsl). The first 12 volumes were discarded to allow for T1 equilibration effects. Given our hypothesis and the identical temporal design of the MI and EM task, no distinction was made between tasks until the final stage of processing. As 14 subjects (one subject was excluded see below) performed 2 tasks, MI and EM, 28 “blinded” tasks were processed-we use the term blinded as no distinction was made between either imagery or EM during the generation of the IC.

The following data pre-processing steps were applied to the 28 blinded tasks: masking of non-brain voxels; voxel-wise de-meaning of the data; normalization of the voxel-wise variance. No subject moved more than 2 mm. Pre-processed data were whitened and projected into a 52-dimensional subspace using probabilistic Principal Component Analysis where the number of dimensions was estimated using the Laplace approximation to the Bayesian evidence of the model order (Beckmann and Smith, 2004). The whitened observations were decomposed into sets of vectors which describe signal variation across the temporal domain (time-courses), the session/subject domain and across the spatial domain (maps) by optimizing for non-Gaussian spatial source distributions using a fixed-point iteration technique (Hyvarinen, 1999). Estimated Component maps were divided by the standard deviation of the residual noise and thresholded by fitting a mixture model to the histogram of intensity values (Beckmann and Smith, 2004). The time course of each Independent Component was then entered into a general linear model of the convolved block design of Task vs. Rest.

Overall this produces a standard subject score for each IC that incorporates the effect size for each of the 28 blinded task (14 subjects, EM and MI) for the associated spatio-temporal process shown in the spatial map and the time course. An IC was considered to be involved in MI or EM if a one-way *t*-test found it to be significantly different to zero across subjects. If an IC was significantly involved in both tasks then a paired *t*-test was performed on the subject score for each task, i.e., MI and EM.

## Results

### Behavioral results

One subject was excluded because of a failure to perform MI satisfactorily. The remaining 14 subjects performed adequately on all aspects of the hand rotation task (Mean = 95.3%; *SD* = 4.1%), fMRI simulation (Component 2) and Fitts law [mean break point 19% (*SD* = 14.2) less for MI than EM], as well as during the fMRI session. No subject failed to either suppress movement or showed evidence of non-compliance during the fMRI paradigm. Median post-MRI MI scores was 6 (range 4–7).

### fMRI data

#### Whole brain analysis

Fifty-two IC were defined by TICA. IC's that identified artifact recognized by previously published patterns and high frequency were excluded by visual inspection (Beckmann and Smith, 2005). Components that were driven by outliers or were not significant (*p* < 0.01) across task were excluded. Components in which the subject scores were significantly different to zero (for either EM or MI) were included. Seven IC remained.

**Figure 1:**
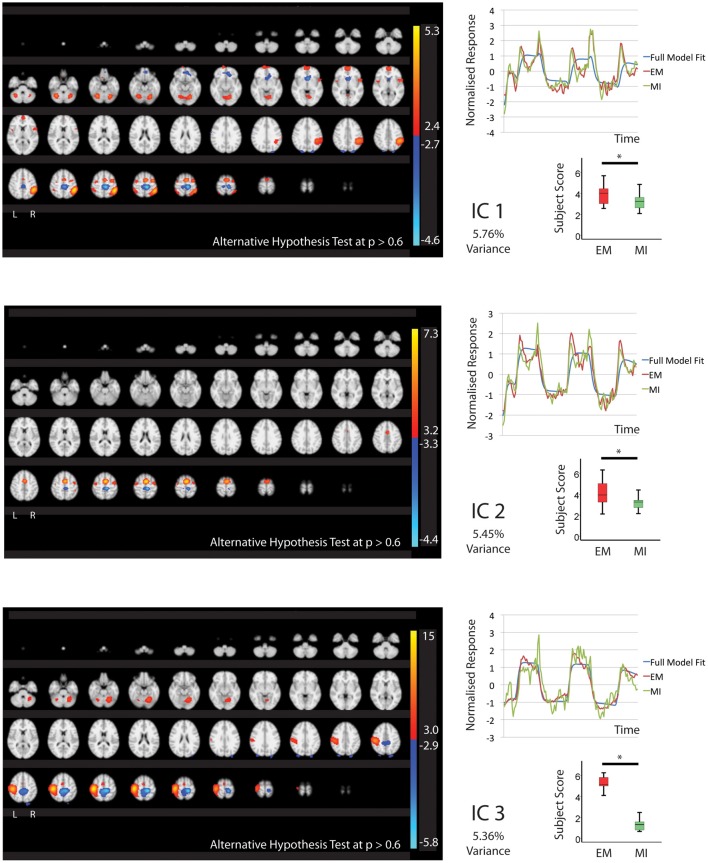
**The figures show the involvement of each IC across the whole brain with a standard threshold of *p* > 0.6 (alternative Hypothesis test) and the variance is accounts for out of the total explained variance**. The scales show the transformed *z*-score, orange is activation, blue is deactivation. The normalized time course response is shown for each task and the full model fit (Full model fit = blue, executed movement = red, motor imagery = green). The mean subject scores with standard error bars are shown for each task and differences highlighted (executed movement = red, motor imagery = green). The IC's (1, 2, 3) that are shared between executed movement and motor imagery. The time course and subject score for each task are shown. ^*^IC1; *p* < 0.01, IC2; *p* < 0.05, IC3; *p* < 0.001.

**Figure 2:**
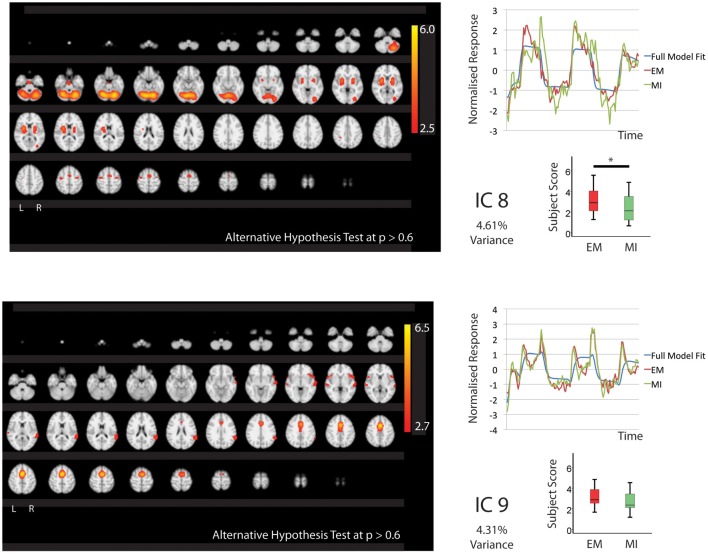
**The figures show the involvement of each IC across the whole brain with a standard threshold of *p* > 0.6 (alternative Hypothesis test) and the variance is accounts for out of the total explained variance**. The scales show the transformed *z*-score, orange is activation, blue is deactivation. The normalized time course response is shown for each task and the full model fit (Full model fit = blue, executed movement = red, motor imagery = green). The IC's (8, 9) that are shared between executed movement and motor imagery. The time course and subject score for each task are shown. ^*^IC8; *p* < 0.05.

**Figure 3:**
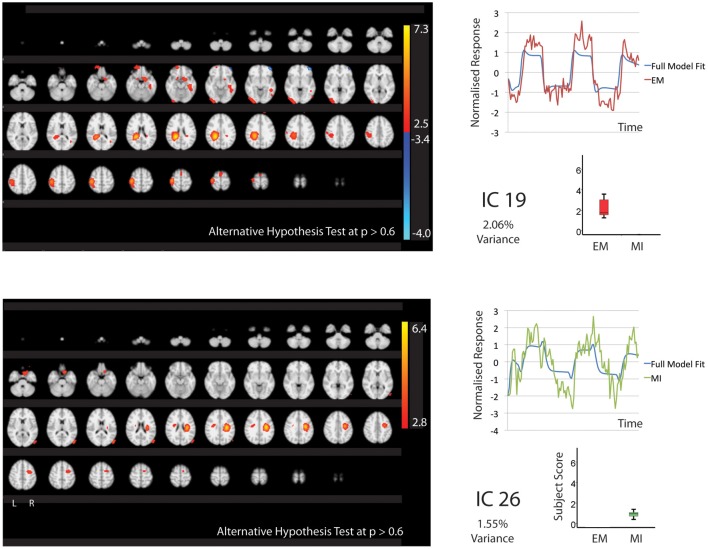
**The figures show the involvement of each IC across the whole brain with a standard threshold of *p* > 0.6 (alternative Hypothesis test) and the variance is accounts for out of the total explained variance**. The scales show the transformed *z*-score, orange is activation, blue is deactivation. The normalized time course response is shown for each task and the full model fit (Full model fit = blue, executed movement = red, motor imagery = green). IC 19 that is related to executed movement only and IC26 that is related to motor imagery only. The time course and subject score for each task are shown.

In keeping with our hypothesis there were IC's that are shared between EM and MI (subject scores significantly greater than zero for both tasks) and components that are exclusive to EM (subject score greater than zero for EM only) and to MI (subject score greater than zero for MI only). The whole brain activations and deactivations, time course, variance explained and subject score are shown in Figures 1–3. Table 1 summarizes the areas involved in each component which were labeled using the Juelich Atlas (Eickhoff et al., 2005). We have previously explored the differential involvement of BA4a and BA4p (subdivisions of the primary motor cortex) in MI and EM (Sharma et al., 2008); given the degree of smoothing required for TICA it was not appropriate consider this areas separately in this study.

**Table 1:** **Regions activated in each independent component**.

	**IC's common to both tasks**										**IC related to EM only**		**IC related to MI only**	
	**IC1**		**IC2**		**IC3**		**IC8**		**IC9**		**IC19**		**IC26**	
	**Left**	**Right**	**Left**	**Right**	**Left**	**Right**	**Left**	**Right**	**Left**	**Right**	**Left**	**Right**	**Left**	**Right**
**BA44**	✓	✓							✓	✓				
**BA4**					✓						✓			✓^**^
**Pre-SMA**							✓	✓			✓	✓		
**SMA**	✓	✓	✓	✓	✓	✓	✓	✓	✓	✓				
**PMd**	✓	✓	✓	✓	✓		✓	✓			✓			✓
**Area 1**	✓				✓						✓			
**Area 2**	✓	✓		✓							✓			
**BA3a**					✓									✓^**^
**BA3b**		✓									✓^*^			
**hIP1**		✓												
**hIP2**	✓	✓			✓						✓			
**hIP3**		✓			✓						✓			
**SPL(7A)**	✓	✓												
**SPL(7PC)**	✓	✓												
**lPC(PFt)**					✓						✓			
**IPC(PFm)**		✓								✓				
**IPC(Pga)**		✓												
**IPC(PF)**		✓												
**Thal_premotor**														
**Thal_motor**							✓	✓						
**Thal_Somatosenosry**							✓	✓						
**TE**							✓	✓		✓				
**CB**	✓	✓			✓	✓	✓	✓						

* Exclusively found in IC19;

** Exclusively found in IC26 (Eickhoff et al., 2005).

#### Independent-components shared by executed movement and motor imagery

Five components (IC1, 2, 3, 8, 9 Figures 1, 2) were significantly involved in both EM and MI (subject scores > 0 for both tasks). These components explained 25.49% of the total explained variance. All of the components significantly correlated with the active blocks of the task. In four of the components (IC1, 2, 3, 9), the subjects score was significantly greater during EM than during MI.

IC1 involved activation of all areas of the right parietal lobe (HIP1-3, SPL, IPC) and to a lesser degree the left parietal lobe (hIP2, SPL) as well as the cerebellum (r7L) and BA44 and premotor areas. IC2 showed activation that was largely limited to the premotor areas bilaterally including PMd and SMA. IC3 showed activation that was predominantly localized to the left hemisphere including motor areas (BA4, cerebellum), premotor (PMd, SMA), somatosensory cortex and left parietal areas hIP2-3 and IPC. The activation patterns of IC8 were largely restricted to subcortical structures notable the thalamus (all areas) and cerebellum with limited involvement of the premotor areas.

IC9 is notable as it is equally involved in MI and EM. This IC involves activation of the preSMA, SMA, BA44 and right IPC (PFm).

IC1, IC2, and IC3 all contained areas of deactivation. This generally involved bilateral dorsal BA4. IC1 contained additional deactivation of the left caudate and SPL in IC3.

#### Independent-components involved during executed movement only

One component, IC19 was significantly involved during EM only (2.06% of explained variance). Again this correlated with the motor tasks rather than rest. This involved activation of areas typically seen in movement; the contralateral motor cortex, somatosensory cortex and hIP2&3. IC19 involved deactivation of the left medial frontal gyrus.

#### Independent-components involved during motor imagery only

IC26 was significantly involved during MI only (1.55% of explained variance). This correlated with MI rather than rest. The activation was restricted to the right hemisphere and included the right BA4, premotor and area 3b.

## Discussion

Here we use a data led method to report that MI and EM share cortical networks. The majority of the networks involved in the tasks appear to be shared (accounting 25.49% of the total explained variance). One network was exclusive to EM (accounting for 2.06% of the explained variance) and another was exclusive to MI (accounting for 1.55% of the explained variance). That being said a number of the shared networks are significantly more involved in EM than MI. This provides an important foundation for the use of MI as an alternative means to access the motor system in diseases that limit physical performance such as stroke (Sharma et al., 2006).

We report that EM and MI indeed share the vast majority of networks. A key area that appears to be shared is the contralateral primary motor cortex. In previous studies using mass univariate methods there has been varying reports of its involvement (Gerardin et al., 2000; Hanakawa et al., 2003, 2008; Sharma et al., 2008) for a meta-analysis see (Hetu et al., 2013). On a subset of these subjects we have previously reported that MI activates the posterior division rather than the anterior division of the motor cortex (Sharma et al., 2008). In addition to methodological issues with monitoring MI compliance (see Sharma et al., 2006) we have previously suggested that this may explain the lack of BA4 activation often seen in studies of imagery (Hetu et al., 2013).

The motor cortex is a central node in motor learning (Muellbacher et al., 2002) and recovery after stroke (Calautti et al., 2001; Ward and Cohen, 2004; Cramer, 2008; Sharma and Cohen, 2010). Demonstrating that MI includes the contralateral primary motor cortex strengthens the rationale for using it as a form of training after stroke. The motor cortex has been shown to have a number of different functions (Sanes and Donoghue, 2000). In this context it is likely to be involved in aspects of motor control that precede actual movement (as a result of discharge via the CST). The deactivation of the dorsal aspect of BA4 in IC's 1,2, and 3, needs to be explored further. Consistent with studies using conventional fMRI analysis (Gerardin et al., 2000; Sharma et al., 2008) it should be noted that while IC's involving the contralateral motor cortex are shared between imagery and EM they are more involved in the latter. This raises an important point. Typically MI is used as an alternative means to access the motor system when EM is difficult or not possible (Sharma et al., 2006). Given that we report that the shared networks are activated less during imagery than EM, our results imply that for MI to be as effective as EM the duration of training may need to be greater. Indeed behavioral studies suggest MI training is generally less effective than physical training (Gentili et al., 2010).

The one cortical network that appears to be equally shared between the two tasks involves the supplementary motor cortex (SMA). The SMA been implicated in motor planning and learning (Halsband and Lange, 2006). A previous study has suggested that the role of SMA in MI is to suppress motor output via the motor cortex (Kasess et al., 2008). Although our results to not directly address this point, the observation that the network is equally shared with EM would argue against this view. Effective connectivity of fMRI data has shown that imagery and EM have similar connections (Gao et al., 2011). Indeed studies of the effective connectivity between cortical areas suggests that imagery is capable of highlighting changes not apparent during EM after stroke (Sharma et al., 2009a). There have been numerous studies that use MI to control brain-computer interfaces that typically involve recording from the motor cortex (Wolpaw et al., 2002; Buch et al., 2008, 2012). Although speculative, our results suggest that in principle SMA may be a suitable alternative or additional site for brain computer interfaces (BCI) devices.

It is not surprising that there is a network that is exclusive to EM. Of course the most striking difference between imagery and execution is the discharge via the CST that produces movement and sensory feedback. The cortical areas present in the EM exclusive network involve activation of the contralateral primary motor cortex and the somatosensory cortex. Although the result should not be over interpreted it should be noted that this network is largely restricted to the left hemisphere. Whether this finding would be replicated in similar analysis involving stroke patients would be of interest. It is conceivable that TICA could resolve the debate of whether the bilateral activation often seen after stroke (Calautti et al., 2001; Ward et al., 2003) is related to discharge via the CST or processes that preceed movement. This could be addressed in future studies using similar multivariate analysis.

We report a network that appears exclusive for MI. Typically MI is thought to be a simple surrogate for EM and is often not considered as useful in its own right. Our data further establishes that this is not so. The cortical network involves the ipsilateral motor cortex and BA3a (exclusive to this network) and the ipsilateral PMd (common across networks see Table 1). It has previously been shown that PMd is important to motor recovery after stroke (Calautti et al., 2001), particularly in subjects who are more severely impaired (Johansen-Berg et al., 2002). The role of PMd in these cases may be related to action selection and goal directed movement. Whether MI will have greater beneficial effect in that patient population, i.e., more severely affected remains unknown.

Here we have reported that imagery and EM share a number of key networks. While we have commented upon these networks individually further work is required to understand the interaction between them. It is reasonable to presume that the IC related exclusively to EM occurs during discharge via the CST, but to fully understand the relationship between these networks and the underlying cognitive processes will require methods with much greater temporal resolution for example magnetoencephalography (MEG).

TICA appears to be a useful tool in testing hypothesis that explore shared networks. It has its limitations, however. For instance a central assumption in this work is that the two motor tasks have the same temporal profile. It is entirely possible that cortical networks that have different temporal profiles have been overlooked by this method. However, if that were the case then one would expect those areas to have been highlighted by earlier mass-univariate fMRI studies. Furthermore, a recent report has highlighted TICA may not be as robust as Parallel Factor Analysis (PARAFAC) if there is a possible violation of the assumption of spatial independence (Helwig and Hong, 2013). It should be noted, however, that this report only used a simulated data set. The original description of TICA found it to be more robust on simulated and real data sets than PARAFAC (Beckmann and Smith, 2005).

### Conflict of interest statement

The authors declare that the research was conducted in the absence of any commercial or financial relationships that could be construed as a potential conflict of interest.
